# Two cases of giant peritoneal inclusion cysts requiring treatment after total laparoscopic hysterectomy

**DOI:** 10.1093/jscr/rjaa506

**Published:** 2020-12-17

**Authors:** Katsumi Kozasa, Yuki Takemoto, Takeshi Goto, Mariya Kobayashi, Hitomi Sakaguchi, Sho Fujiwara, Fuyuki Ichikawa, Misako Kuroda, Naoko Komura, Asuka Tanaka, Kumi Masuda, Hirofumi Otsuka, Takeshi Yokoi

**Affiliations:** Department of Obstetrics and Gynecology, Kaizuka City Hospital, Osaka, Japan; Department of Obstetrics and Gynecology, Kaizuka City Hospital, Osaka, Japan; Department of Obstetrics and Gynecology, Kaizuka City Hospital, Osaka, Japan; Department of Obstetrics and Gynecology, Osaka University, Osaka, Japan; Department of Obstetrics and Gynecology, Osaka University, Osaka, Japan; Department of Obstetrics and Gynecology, Kaizuka City Hospital, Osaka, Japan; Department of Obstetrics and Gynecology, Kaizuka City Hospital, Osaka, Japan; Department of Obstetrics and Gynecology, Kaizuka City Hospital, Osaka, Japan; Department of Obstetrics and Gynecology, Kaizuka City Hospital, Osaka, Japan; Department of Obstetrics and Gynecology, Kaizuka City Hospital, Osaka, Japan; Department of Obstetrics and Gynecology, Kaizuka City Hospital, Osaka, Japan; Department of Obstetrics and Gynecology, Kaizuka City Hospital, Osaka, Japan; Department of Obstetrics and Gynecology, Kaizuka City Hospital, Osaka, Japan

**Keywords:** peritoneal inclusion cyst, PIC, pseudocyst, adhesion, total laparoscopic hysterectomy, TLH

## Abstract

Peritoneal inclusion cysts (PICs) often develop in post-operative patients. Since the incidence of adhesions is lower with laparoscopic surgery than with open surgery, PICs are less likely to occur in the former. Although post-operative adhesions or PICs rarely develop after laparoscopic surgery (such as total laparoscopic hysterectomy: TLH), we encountered two cases of giant PICs with abdominal pain after TLH. In Case 1, strong adhesion was already present when TLH was performed. Therefore, this case may have been predisposed to the development of adhesions in the abdominal cavity. However, no adhesions were observed during TLH in case 2, and there were no risk factors, such as pre-operative adhesions and endometriosis. Therefore, adhesions and PICs may develop even after TLH, and approaches need to be considered for their prevention.

## INTRODUCTION

Adhesions are one of the most important long-term complications of abdominal surgery. They occur in 79–90% of patients undergoing laparotomy. Laparoscopic surgery reduces the degree and severity of adhesion formation by approximately 50% (1).

Laparoscopic surgery for hysterectomy (total laparoscopic hysterectomy: TLH) results in fewer post-operative adhesions than open surgery (total abdominal hysterectomy: TAH). Peritoneal inclusion cysts (PICs) are cyst-like structures containing ascitic fluid that accumulated in closed cavities caused by adhesions due to surgery, infection and endometriosis. Therefore, the incidence of PICs may be lower with TLH than with TAH. There is currently no information on the development of PICs after TLH. We herein report two cases of giant PICs with symptoms that developed after TLH.

## CASE REPORT

### Case 1

**Figure 1:**
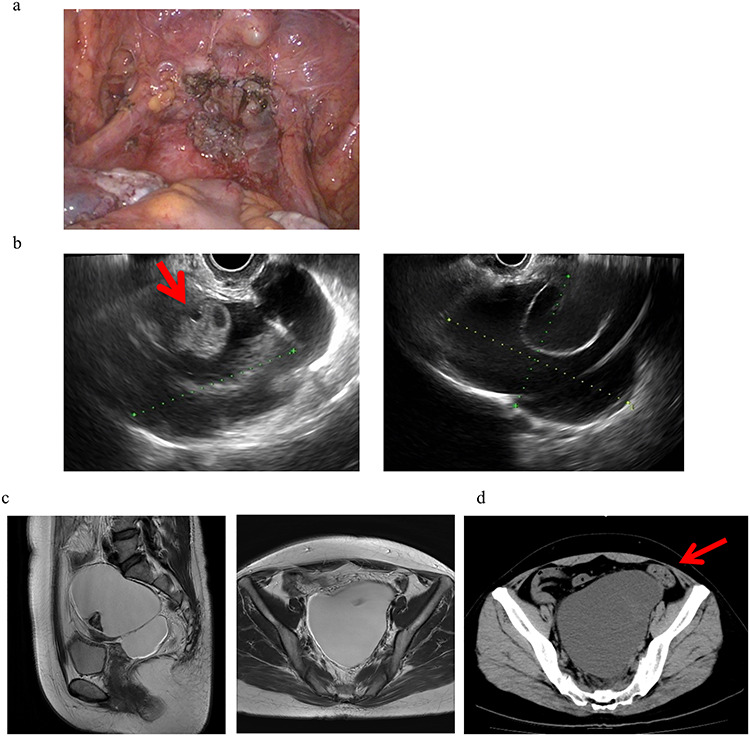
Clinical findings of case 1. (**a**) Findings at the end of TLH. No abnormal findings were detected. (**b**) An US image of PIC in case 1. An ovary with a normal appearance was observed within the multilocular cyst. (**c**) T2-weighted MRI image. (**d**) CT findings with abdominal pain. A giant cyst appears to compress the sigmoid colon, resulting in abdominal pain and the impaired passage of the stool mass.

A 46-year-old woman, gravida 0 was diagnosed with uterine myoma. TLH was performed for symptom relief. As an intra-operative finding, strong adhesion was observed between the posterior wall of the uterus and the intestinal tract in the Douglas fossa, which led to difficulties detaching the adhesion. At the end of surgery, no abnormal findings, such as adhesions, were detected, as shown in Fig. 1a. The pathological diagnosis was uterine myoma with no evidence of endometriosis. Three months after surgery, a 13-cm cyst was detected on the vaginal stump by transvaginal ultrasound (US) in a post-operative follow-up (Fig. 1b). Pelvic magnetic resonance imaging (MRI) revealed PIC that was asymptomatic and, thus, no treatment was initiated (Fig. 1c).

Eight months after surgery, the patient presented with lower abdominal pain and constipation. Computerized tomographic (CT) images showed a giant cyst compressing the sigmoid colon, resulting in abdominal pain and the impaired passage of the stool mass (Fig. 1d). To attenuate these symptoms, the cyst was punctured and ~700 ml of a dark brown smooth liquid was aspirated transvaginally, which attenuated her symptoms. The cyst subsequently increased in size to 12 cm but was asymptomatic and thus is being followed up without treatment.

### CASE 2

**Figure 2:**
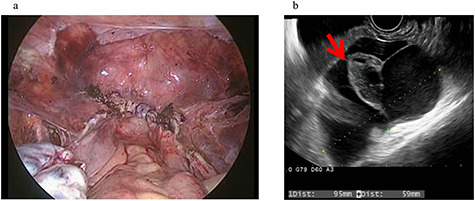
Clinical findings of case 2 (before surgery for PIC)**.** (**a**) Findings at the end of TLH. No abnormal findings were detected. (**b**) An US image of PIC. The major axis was 95 mm. An ovary with a normal appearance was observed within the multilocular cyst.

A 43-year-old woman, gravida 2, para 2 was diagnosed with uterine myoma and underwent TLH. No abnormal intra-abdominal findings, such as adhesions, endometriosis or ovarian cysts, were detected. As shown in Fig. 2a, no abnormal findings, such as adhesions, were detected at the end of surgery or 2 months later.

**Figure 3:**
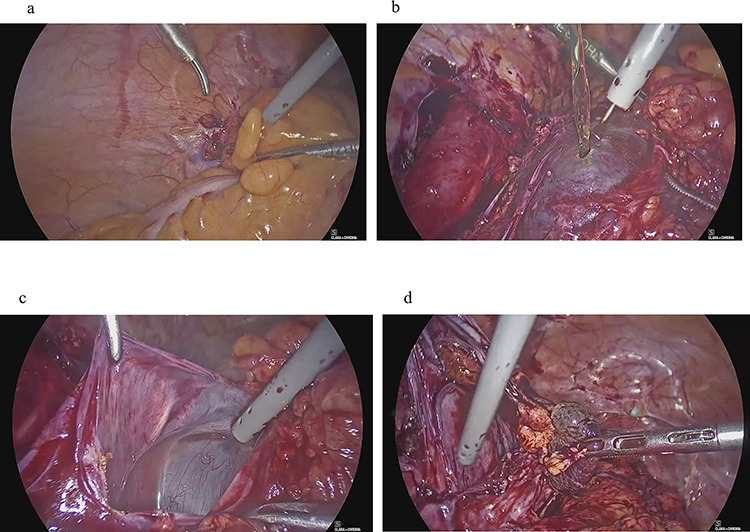
Clinical findings of case 2 (during surgery for PIC)**.** (**a**) Strong adhesion between the sigmoid colon and pelvic wall in the left adnexal region. (**b**) When the sigmoid mesentery and left pelvic peritoneum were removed and the cyst was opened, a yellow transparent liquid was ejected. (**c**) The cavity formed by adhesions. (**d**) Left ovarian tissue inside PIC.

Four years and 6 months after surgery, she presented with lower left abdominal pain. A multilocular cyst, measuring ~10 cm, was detected on the vaginal stump by transvaginal US (Fig. 2b). Since the cyst was considered to be causing her symptoms, emergency surgery was performed. Adhesions were observed in the left adnexal region, and a clear yellow liquid was retained in the cavity formed by the adhesions (Fig. 3). The adhesions were removed by laparoscopic surgery and left adnexal excision was performed, which ameliorated her symptoms.

## DISCUSSION

PICs are formed by closed cavities caused by adhesions. They are formed by the differentiation of mesothelial cells, which repairs the damaged part of the peritoneum. Usually these cysts are closed spaces between adhesions with entrapped fluid, and sometimes if they are long standing, they will get epithelialized, and epithelial lining will produce fluid leading the cysts to keep increasing in size. They typically occur in women. In a study of 228 PICs, 82.5% of cases were female (2). The development of PICs involves the production of ascites by the ovaries, hemorrhage during ovulation, and fluid produced by the ovaries in the follicle and luteal phases, and thus, the incidence of PICs is high in women. In a previous study on 31 PICs, those in 84% of cases were external to or entrapped the ipsilateral ovary. Therefore, the ovaries are involved in the development of PICs (3). Pre-operative adhesions and endometriosis are involved in the onset of PICs, as in case 1; however, these risk factors were absent in case 2. Post-operative adhesions alone can cause PICs.

A feasible management option for asymptomatic patients with incidentally discovered PICs involves observations with serial imaging. When surgery is performed, the risk of local recurrence is 30–50% (4). Oophorectomy at the time of primary surgical resection appears to be beneficial for reducing the recurrence rate; however, a concrete conclusion cannot yet be reached. Based on the presenting symptoms in case 2, adhesiotomy and left ovariectomy were performed, which attenuated her symptoms. Since functioning ovaries are involved in the development of PICs, hormone therapy may be effective. Oral contraceptives (OCs) may shrink the size of PICs and attenuate symptoms by decreasing the production of cyst fluid (5). However, the discontinuation of OCs may lead to recurrence via the re-accumulation of peritoneal fluid (4). PICs disappeared in 7 out of 8 cases treated with GnRH, demonstrating its efficacy. However, two patients relapsed after the end of treatment (6). Image-guided aspiration may provide fluid for a cytological examination and resolve symptoms with minimal intervention. Recurrence was previously reported to be common in patients who were treated with aspiration only (4). However, ovarian fluid production was often suppressed by aspiration in combination with OC (4). In a small series of cases, US-guided aspiration with GnRH agonist therapy prevented recurrence (7).

Fewer adhesions occur with laparoscopy than with open surgery (1). The rate of rehospitalization due to disorders directly related to adhesions within 5 years of surgery was significantly lower with laparoscopic (1.7%) than with open surgery (4.3%). Regarding hysterectomy, tissue damage was significantly less with TLH than with TAH, indicating fewer postoperative adhesions (8–10). PICs are rarely detected after TLH, and to the best of our knowledge, there is currently no information on PICs after TLH. Among 517 cases that underwent TLH at our hospital over 5 years, two developed PICs that required treatment. However, asymptomatic patients have not been to the hospital; the number of patients with PICs may have been higher. Post-operative adhesions and their prevention need to be considered, even in TLH. When pelvic cysts are detected after hysterectomy, treatment strategies need to be selected based on the possibility of PICs, even after TLH, without concluding they are ovarian tumours.
